# WEB downloadable software for training in cardiovascular hemodynamics in the (3-D) stress echo lab

**DOI:** 10.1186/1476-7120-8-48

**Published:** 2010-11-13

**Authors:** Tonino Bombardini, Davide Cini, Giorgio Arpesella, Eugenio Picano

**Affiliations:** 1 Institute of Clinical Physiology, CNR, Pisa, Italy; 2 Department of Surgery and Transplants, University of Bologna, Italy

## Abstract

When a physiological (exercise) stress echo is scheduled, interest focuses on wall motion segmental contraction abnormalities to diagnose ischemic response to stress, and on left ventricular ejection fraction to assess contractile reserve. Echocardiographic evaluation of volumes (plus standard assessment of heart rate and blood pressure) is ideally suited for the quantitative and accurate calculation of a set of parameters allowing a complete characterization of cardiovascular hemodynamics (including cardiac output and systemic vascular resistance), left ventricular elastance (mirroring left ventricular contractility, theoretically independent of preload and afterload changes heavily affecting the ejection fraction), arterial elastance, ventricular arterial coupling (a central determinant of net cardiovascular performance in normal and pathological conditions), and diastolic function (through the diastolic mean filling rate). All these parameters were previously inaccessible, inaccurate or labor-intensive and now become, at least in principle, available in the stress echocardiography laboratory since all of them need an accurate estimation of left ventricular volumes and stroke volume, easily derived from 3 D echo.

Aims of this paper are: 1) to propose a simple method to assess a set of parameters allowing a complete characterization of cardiovascular hemodynamics in the stress echo lab, from basic measurements to calculations 2) to propose a simple, web-based software program, to learn and training calculations as a phantom of the everyday activity in the busy stress echo lab 3) to show examples of software testing in a way that proves its value.

The informatics infrastructure is available on the web, linking to http://cctrainer.ifc.cnr.it

## Introduction

Recent technological development and engineering refinements have allowed the application of real-time three-dimensional (RT3D) echocardiography in the routine clinical setting [1]. Because full-volume datasets obtained with RT3 D echocardiography incorporate information on the entire left ventricle in four volumetric datasets, RT3 D echocardiography has the potential to overcome many of the limitations encountered with two-dimensional echocardiography, mostly by eliminating the need for geometric modelling and the errors caused by the use of foreshortened views [2]. Three-dimensional (3D) volumetric imaging has potential advantages in stress echocardiography, including the ability to provides an accurate assessment of stroke volume, allowing derivation of a set of hemodynamic measures usually difficult or impossible to obtain with two-dimensional (2D) echocardiography [3-6]. However calculations are time-consuming and difficult to plan in the busy stress echo lab, and generally cardiologists are not well-trained in these calculations. Therefore we present a WEB based software program for calculation training in the 3 D stress echo lab. The "algorithms", "testing" and "implementation" of this new computational method will be described. The informatics infrastructure is available on the web, linking to http://cctrainer.ifc.cnr.it.

**Figure 1:**
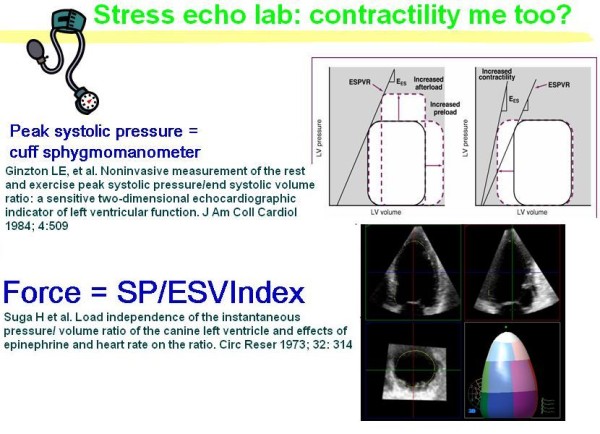
**Stress echo lab: contractility me too?**. *Blood pressure analysis*. One investigator records all blood pressures at rest and during exercise during the study. The blood pressure recording is made using a manometer sphygmomanometer and the diaphragm of a standard stethoscope. *Echocardiography *is performed using 3-D or conventional 2-D echocardiography and left ventricular end-systolic volume is measured. The contractility is determined at each stress step as the ratio of the systolic pressure (cuff sphygmomanometer)/end-systolic volume index (end-systolic volume/body surface area). Modified from Bombardini T. Myocardial contractility in the echo lab: molecular, cellular and pathophysiological basis. Cardiovascular ultrasound 2005, 3:27 [11].

**Figure 2:**
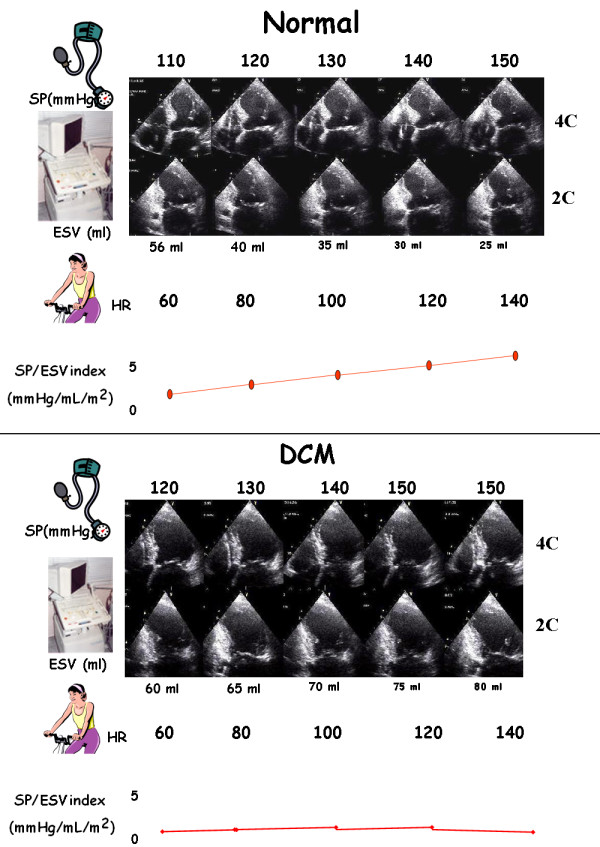
**Contractility changes with heart rate changes**. The force-frequency relationship is defined up-sloping (upper panel) when the peak exercise SP/ESV index is higher than baseline and intermediate stress values; flat or negative (lower panel), when the peak exercise systolic pressure/end-systolic volume index is equal to or lower than baseline stress values. The critical heart rate (or optimum stimulation frequency) is defined as the heart rate at which systolic pressure/end-systolic volume index reaches the maximum value during progressive increase in heart rate.

**Figure 3:**
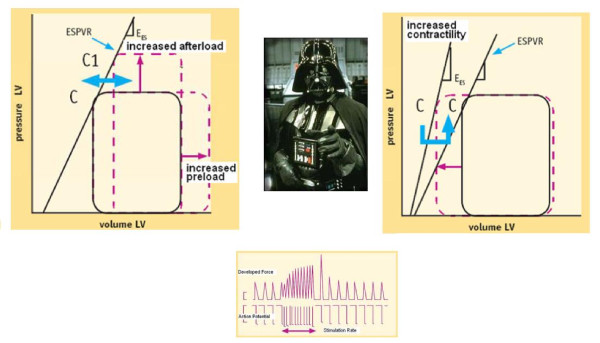
**Preload, afterload, heart rate: the dark side of the force**. When a stress echo is scheduled, interest is focused on wall motion segmental contraction abnormality to diagnose ischemic response to stress and on LVEF to assess contractile reserve. However, ejection fraction is a very gross index of left ventricular performance. It is affected by pre-load and after-load changes and heart rate. *Left panel*. The graph shows how two additional pressure-volume loops appear with an acute increase in afterload or preload. Contractility is quantified by the end-systolic pressure volume relation slope: the *E*es (end systolic elastance). *Right panel*. Increased contractility is reflected in higher myocardial fiber shortening velocity, with a more highly developed tension peak and a steeper pressure rise, when preload, after load, and heart rate are constant: the *E*es (end systolic elastance) moves upward and to the left. *Lower panel*. Force-frequency relation or Bowditch treppe. In the healthy heart, a frequency increase up to 180 beats per minute provides for faster systolic calcium SR release (increased contractility or developed force) and for faster diastolic SR calcium reuptake (positive lusitropic effect).

**Figure 4:**
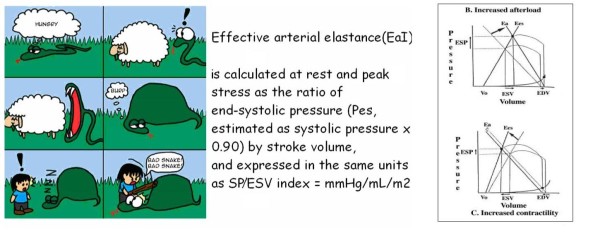
**Arterial elastance**. In the cartoon (left panel) a compliant and no resistive young hungry snake eats a big sheep easily. In the cardiovascular system the hydraulic load, namely effective arterial elastance, is described by the formula: End Systolic Pressure/Stroke Volume/Body Surface Area (right panel). Intuitively a well compliant and no resistive vascular system easily accomplish a big stroke volume without great increase of systemic pressure: steady End Systolic Pressure/Stroke Volume ratio; "low-good" arterial elastance. Intuitively a bad compliant and resistive vascular system difficultly accomplish the stroke volume with greater increase of systemic pressure: increased End Systolic Pressure/Stroke Volume ratio; "high-bad" arterial elastance. On the basis of the windkessel model, effective arterial elastance *E*_a _is a steady-state arterial parameter that incorporates peripheral resistance, characteristic impedance, and total lumped arterial compliance and that also incorporates systolic and diastolic time intervals. Since the pioneer work of Kelly et al. [38], which confirmed the clinical applicability of this concept in humans, effective arterial elastance *E*_a _has been used to quantify arterial load during aging, in hypertensive patients, and in various forms of cardiac disease. *Ees *= left ventricular end-systolic elastance, ESV = end-systolic volume, ESP = end-systolic pressure, EDV = end-diastolic volume.

**Figure 5:**
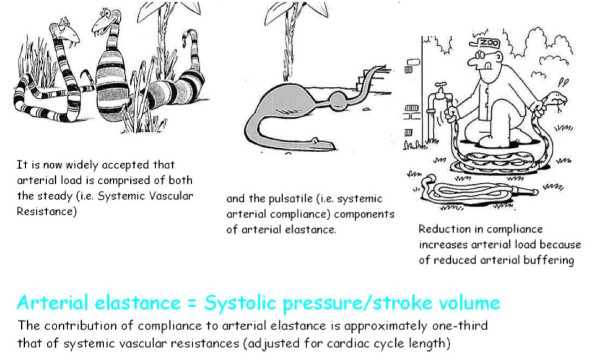
**The steady (Systemic Vascular Resistance) and the pulsatile (Systemic Arterial Compliance) components of Arterial Elastance**. In the cartoon (left panel) the skinny snake is a thin pipe with high strength, the snake stretched by food is a low resistance conduit. In the cartoon (middle panel) the compliant snake easily dilates itself and pushes the food at pulsatile waves. In the cartoon (right panel) the blind zoo guardian unsuccessfully try to use the snake tail (extremely resistive and no compliant) as a water conduct. Increased systemic vascular resistance and decreased total arterial compliance both contribute to the high arterial load in hypertensive patients.

**Figure 6:**
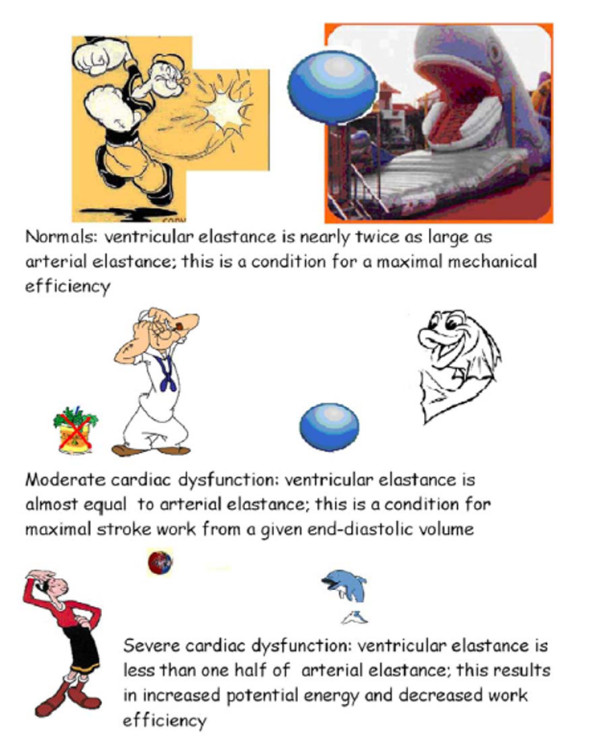
**Ventricular-arterial coupling**. In the cartoon, arterial elastance is the "mouth" of the whale. Big mouth means low arterial elastance (no resistive and well compliant), able to easily accommodate large stroke volume energetically ejected; small fish mouth means higher arterial elastance (highly resistive and no compliant), able to accommodate only small stroke volume (low contractile-energy launched). Understanding the performance of the left ventricle (LV) requires not only examining the properties of the LV itself, but also investigating the modulating effects of the arterial system on left ventricular performance. Interaction of the LV with the arterial system, termed ventricular-arterial coupling, is a central determinant of cardiovascular performance and cardiac energetics. Ventricular arterial coupling is indexed by the ratio of left ventricular systolic elastance index (systolic pressure/end-systolic volume) to arterial elastance (*E*a, ratio of end-systolic pressure by stroke volume). Although in the resting state ventricular-arterial coupling is maintained in a range that maximizes the efficiency of the heart, when the system is stressed, energy efficiency is sacrificed in favor of cardiac efficiency, manifested by an increase in the coupling index (i.e., a greater relative increase in ventricular contractility than arterial load). Ventricular arterial coupling is normally set toward higher left ventricular work efficiency, whereas in patients with moderate cardiac dysfunction, ventricular and arterial properties are matched to maximize stroke work at the expense of work efficiency.

Once the electronic rest and stress data set is filled, the CCtrainer program will show that echo-measured volumes (plus standard assessment of heart rate and blood pressure) allow a complete characterization of cardiovascular hemodynamics (including cardiac output and systemic vascular resistance), left ventricular elastance (Figure 1, Figure 2, Figure 3) arterial elastance (essential for characterizing the distal impedance of the arterial system downstream of the aortic valve) (Figure 4, Figure 5), ventricular arterial coupling, a central determinant of net cardiovascular performance in normal and pathological conditions (Figure 6), and diastolic function [7-11].

## Methods

### Web-based system architecture

A computerized web-based system has been developed for data transfer, analysis and report phases. All the computer clients connect to the server merely by using a common web browser, without needing to install any specific software. Data and reports are anonymous, so secure protocols are not required.

### Communication phases

The informatics infrastructure is available on the web, linking to http://cctrainer.ifc.cnr.it.

When operating for the first time, a user need to get his/her user - password clicking on the "new user" button. The CCTRAINER calculation program is completely free for use during the first month of operations. Algorithms and data sheets can be freely downloaded. Values and indexes are obtained automatically by software processing. After the first month, to continue using the program each user must be registered by the server administrator who assigns him/her an account and an exclusive memory area on the database server. A user license could be purchased by bank transfer to the CNR. Page 1 of the WEB site http://cctrainer.ifc.cnr.it. gives time and modalities of extending the time of program use. Only then can the user access his/her data in protected and reserved mode.

### Parameters to be filled in on the "rest and peak stress" electronic sheet

In each training example the age, height, and weight will be filled in and body mass index and body surface area will be calculated. A complete data set to fill in the electronic sheet is shown in Table 1 and listed below:

**Table 1:** Rest and peak stress data set

Values	Method	*Measure unit*
**Height**	Standard	*cm*
**Weight**	Standard	*kg*
**Heart rate**	ECG	*bpm*
***"Left" heart***		
**LV ESV**	3 D echo or 2 D echo (Simpson rule)	*mL*
**LV EDV**	3 D echo or 2 D echo (Simpson rule)	*mL*
**SBP**	Sphygmomanometer or Tonometer	*mmHg*
**DBP**	Sphygmomanometer or Tonometer	*mmHg*
**Mitral E**	Doppler	*cm/sec*
**Mitral TDI e´**	Tissue Doppler	*cm/sec*
**Diastolic time**	M-Mode or Precordial Sensor	*msec*
**Diastolic time/systolic time ratio**	M-Mode or Sensor	*ratio*
***"right"heart***		
**Peak TR jet velocity**	Doppler	*m/sec*
**TR peak gradient**	Doppler	*mmHg*
**Right Atrium Pressure (RA pressure)**	2 D echo - vena cava respiratory motion	*mmHg*
**Pulmonary artery end-diastolic velocity**	Doppler	*m/sec*
**Early pulmonary regurgitation velocity**	Doppler	*m/sec*
**Right Ventricular Outflow Tract (RVOT) Time-Velocity Integral (TVI)**	Doppler	*cm*

SBP = Systolic Blood Pressure; DBP = Diastolic Blood Pressure; TR = Tricuspid Regurgitation; LV EDV = Left Ventricular End-Diastolic Volume; LV ESV = Left Ventricular End-Systolic Volume; RV = Right Ventricular

#### Heart rate (bpm)

##### Systolic Blood Pressure (SBP, mmHg) and Diastolic Blood Pressure (DBP, mmHg)

The blood pressure recording is made using a sphygmomanometer and the diaphragm of a standard stethoscope. The auscultatory pressure is obtained by inflating the cuff to a level approximately 30 mm Hg above the peak level of systolic blood pressure and evaluating Korotkoff's sounds. The systolic blood pressure is recorded as the point where the first tapping sound occurs for 2 consecutive beats. The right and left brachial arterial systolic blood pressures are compared and discrepancies greater than 5 mm Hg are reported [12].

**LV End-Diastolic Volume (EDV, mL) **is measured from the apical view by an experienced observer using 3 D echo or by apical 4- and 2-chamber views using (Simpson's rule) 2 D echo. The frame captured at the R-wave in ECG in triggered software is considered to be the end-diastolic frame [13,14]; or the end-diastolic volume is obtained from the LV volume curves as the maximum value.

**LV End-Systolic Volume (ESV mL) **is measured from the apical view by an experienced observer using 3 D echo or by apical 4- and 2-chamber views using (Simpson's rule) 2 D echo. The frame captured at the smallest left ventricular cavity is considered to be the end-systolic frame [13,14].

##### Pulsed Doppler mitral diastolic E (cm/sec)

Pulsed Doppler mitral diastolic inflow (E, cm/sec) is recorded using pulse-wave Doppler, with the sample volume placed at the tips of the mitral leaflets on the apical four-chamber view. Mitral inflow velocity measurements are recorded at end-expiration during ambient respiration [15].

##### Tissue Doppler mitral annular tissue velocity e' (cm/sec)

Mitral annular tissue velocity (e', cm/sec) is recorded using pulse-wave Doppler in the apical four-chamber view. It is recommended to acquire and measure tissue Doppler signals at least at the septal and lateral sides of the mitral annulus and their average, given the influence of regional function on these velocities and time intervals. Mitral annular tissue velocity measurements are recorded at end-expiration during ambient respiration [15].

##### Diastolic time (M-Mode or Precordial Sensor, msec)

Prior work concerning diastolic duration (diastolic time) has been motivated primarily by consideration of diastolic myocardial perfusion time rather than the duration of mechanical events, and has been used to assess the heart rate-dependent effects of pharmacological agents [16,17].

**Figure 7:**
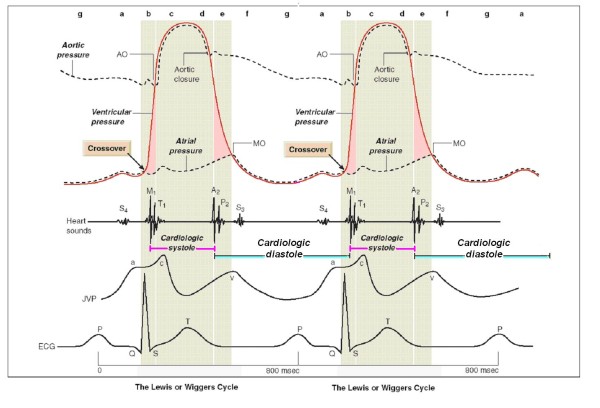
**The mechanical events in the cardiac cycle, assembled by Lewis in 1920 but first conceived by Wiggers in 1915**. Cycle length of 800 milliseconds for 75 beats/min. *Cardiological systole *is demarcated by the interval between the first and the second heart sounds, lasting from the first heart sound to the closure of the aortic valve. The remainder of the cardiac cycle automatically becomes *cardiological diastole*. Left ventricular contraction: isovolumic contraction (b); maximal ejection (c). Let ventricular relaxation: start of relaxation and reduced ejection (d); isovolumic relaxation (e); LV filling rapid phase (f); slow LV filling (diastasis) (g); atrial systole or booster (a). Mitral valve closure occurs *after *the crossover point of atrial and ventricular pressures at the start of systole. A_2 _= aortic valve closure, aortic component of second sound; AO = aortic valve opening, normally inaudible; ECG = electrocardiogram; JVP = jugular venous pressure; M_1 _= mitral component of first sound at time of mitral valve closure; MO = mitral valve opening, may be audible in mitral stenosis as the opening snap; P_2 _= pulmonary component of second sound, pulmonary valve closure; S_3 _= third heart sound; S_4 _= fourth heart sound; T_1 _= tricuspid valve closure, second component of first heart sound. Modified from Opie LH. Mechanisms of cardiac contraction and relaxation. In: Braunwald E, Zipes DP, Libby P, Bonow RO, eds. Heart Disease. 7^th ^ed. WB Saunders Company 2005, Chap.19:457-489, page 475.

Recent studies utilizing both the exercise radionuclide angiography time activity curve [18] or Doppler echocardiography [19,20] assessed that cardiac performance may be characterized in terms of the relative duration of diastole and systole. Cardiac cycle abnormalities of patients with heart failure are characterized by a prolongation of left ventricular systole and an abnormal shortening of left ventricular diastole [21]. A practical approach is to calculate cardiological diastolic and systolic time in the echo lab (Figure 7) (Table 2).

**Table 2:** Physiological vs cardiological systole and diastole

Physiological systole	Cardiological systole
Isovolumic contraction	From M_1 _to A_2_, including:
Maximal ejection	
	Major part of isovolumic contraction*
	Maximal ejection
	Reduced ejection
**Physiological diastole**	**Cardiological diastole**
Reduced ejection	A_2 _- M_1 _interval (filling phases included)
Isovolumic relaxation	
Filling phases	

In Greek, *systole *means contraction and *diastole *means "to send apart". *Physiological systole *lasts from the start of isovolumic contraction to the peak of the ejection phase, so that physiological diastole commences as the LV pressure starts to fall. This concept fits well with the standard pressure-volume curve. *Physiological diastole *commences as calcium ions are taken up into the SR, so that the myocyte relaxation dominates over contraction and the LV pressure starts to fall as shown on the pressure-volume curve. In contrast, *cardiological systole *is demarcated by the interval between the first and the second heart sounds, lasting from the first heart sound to the closure of the aortic valve. The remainder of the cardiac cycle automatically becomes *cardiological diastole*.

* Note that M_1 _occurs with a definite albeit short delay after the start of the LV contraction. Modified from Opie LH. Mechanisms of cardiac contraction and relaxation. In: Braunwald E, Zipes DP, Libby P, Bonow RO, eds. Heart Disease. 7^th ^ed. WB Saunders Company 2005, Chap.19:457-489, page 474.

**Figure 8:**
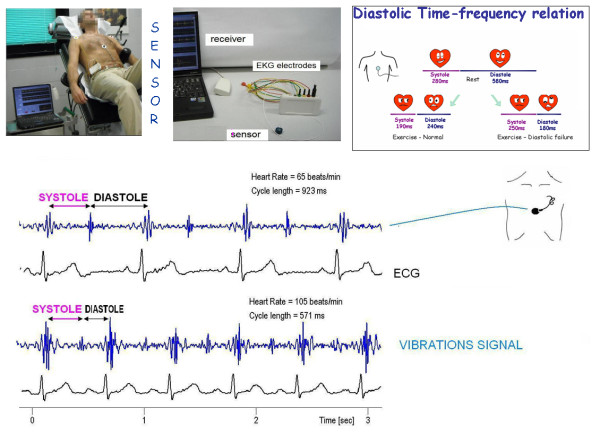
**Operator-independent cardiologic systole and diastole quantification**. The transcutaneous force sensor is based on a linear accelerometer. We housed the device in a small case which is positioned in the mid-sternal precordial region and is fastened by a solid gel ECG electrode. The acceleration signal is converted to digital and recorded by a laptop PC, together with an ECG signal. An analog peak-to-peak detector synchronized with the standard ECG scans the first 150 ms following the R wave to record first heart sound force vibrations and the 100 ms following the T wave to record second heart sound force vibrations. A stable, reproducible, and consistent first heart sound and second heart sound signal is obtained and utilized as time markers to continuously assess cardiologic systole and diastole during exercise, or pharmacological stress echo. Modified from Bombardini *et al*. *Cardiovascular Ultrasound *2008 6:15 [24]

A cutaneous operator independent force sensor, based on first heart and second heart sound vibrations amplitude recording [22,23], may be utilized to automatically quantify cardiological systole and cardiological diastole duration [24] (Table 2) (Figure 8). Simultaneous calculation of stroke volume allows quantifying diastolic filling rate and its changes with stress.

##### Diastolic time/systolic time ratio

The diastolic/systolic time ratio is expressed as a dimensionless ratio (diastolic/systolic, normal value > 1). Reversal of the normal diastolic/systolic ratio may compromise cardiac filling and function. The diastolic/systolic mismatch is accentuated during exercise and has the potential to impair the cardiac reserve in these patients by restricting ventricular filling and coronary perfusion time [25] (Table 3).

**Table 3:** Heart rate and total cardiac cycle duration

HR b.p.m	50	60	70	80	90	100	110	120	130	140	150	160	170	180
Cardiac cycle length (msec)	1200	1000	857	750	666	600	545	500	463	429	400	375	353	333

The total cardiac cycle duration is algebraically dependent on the heart rate [= 60,000 msec/heart rate] with fixed values totally independent from the increasing heart rate stress type. However, at each heart rate the fixed total cardiac cycle time can be differently divided between systole and diastole. At fixed heart rates the diastolic time fraction is determined by factors that modulate systolic duration through modulation of myocyte contraction.

##### Tricuspid Regurgitation Peak Gradient (TRPG, mmHg)

Tricuspid regurgitation velocity is usually obtained with continuous wave Doppler from the right ventricular inflow or the apical four-chamber view position. Tricuspid regurgitation velocity reflects the pressure difference during systole between the right ventricle and the right atrium, that is:

$$Transtricuspid\;pressure\;gradient=4*Tricuspid\;regurgitation\;velocity^{2}$$

Normal resting values are usually defined as a peak tricuspid regurgitation velocity of < 2.8 to 2.9 m/s. This value may increase with age and increasing body surface area and this should be considered when estimations are at the upper limits of normal. Further evaluation of patients with dyspnea with estimated right ventricular systolic pressure > 40 mmHg is recommended [26].

**Right Atrial Pressure (RAP, mmHg) **is estimated from the inferior vena cava diameter adjacent to the right atrium in the subcostal view [27] (Table 4). As right atrium pressure increases, this is transmitted to the inferior vena cava, resulting in reduced collapse with inspiration and inferior vena cava dilatation. A inferior vena cava diameter < 2.1 cm that collapses > 50% with a sniff suggests a normal right atrium pressure of 3 mm Hg (range, 0-5 mm Hg), whereas an inferior vena cava diameter > 2.1 cm that collapses < 50% with a sniff suggests a high right atrium pressure of 15 mm Hg (range, 10-20 mm Hg). In indeterminate cases in which the inferior vena cava diameter and collapse do not fit this paradigm (inferior vena cava diameter > 2.1 cm that collapses > 50% with a sniff; or inferior vena cava diameter < 2.1 cm that collapses > 50% with a sniff) an intermediate value of 8 mm Hg (range, 5-10 mm Hg) may be used, or, preferably, secondary indices of elevated right atrium pressure should be integrated [27].

**Table 4:** Estimation of RA pressure based on Inferior Vena Cava diameter and collapse

Inferior vena cava diameter (cm)	Respiratory collapse (%)	RAP (mmHg)
< 2.1	≥ 50%	3 (0-5)
> 2.1	< 50%	15
> 2.1	≥ 50%	8 (5-10)
< 2.1	< 50%	8 (5-10)

RAP = Right atrium pressure

##### Pulmonary Artery end-diastolic velocity (m/sec)

The end-diastolic velocity of pulmonary regurgitation reflects the end-diastolic pressure gradient between the pulmonary artery and the right ventricle. Normally, the pulmonary regurgitation end-diastolic pressure gradient is less than 5 mmHg. An increase in this pressure gradient (> 5 mmHg) has been found to correlate with systolic dysfunction, diastolic dysfunction, increased brain natriuretic peptide, and decreased functional status [28].

##### Peak pulmonary regurgitation velocity (m/sec)

The peak early diastolic pulmonary regurgitation velocity is useful in estimating mean pulmonary artery pressure. The peak diastolic pressure gradient between the pulmonary artery and the right ventricle approximates mean pulmonary artery pressure [29].

##### Time-Velocity Integral (TVI, cm) of Right Ventricular Outflow Tract flow (RVOT)

The right ventricular outflow tract time-velocity integral is obtained by placing a 1- to 2-mm pulsed wave Doppler sample volume in the proximal right ventricular outflow tract just within the pulmonary valve when imaged from the parasternal short-axis view [30].

**Figure 9:**
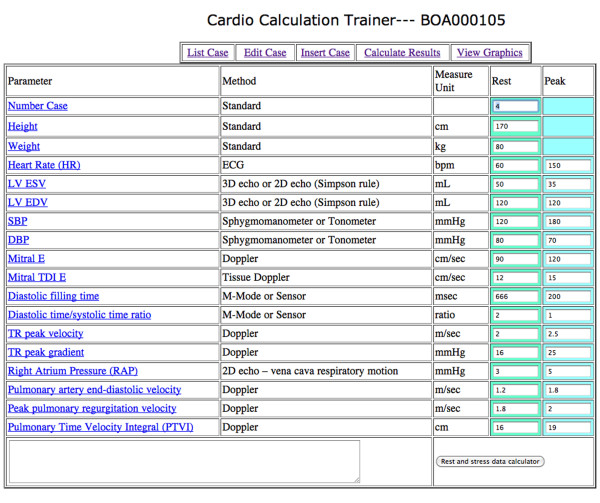
**Implementation of the computational training software**. Opening a new example by the web linking to http://cctrainer.ifc.cnr.it. and clicking on "insert case" button a electronic sheet appears. Rest and peak data sets should be filled by the trainer in the fourth and fifth columns. After inserting the data, the operator clicks the "rest and stress data calculator" button to open the electronic calculation sheet. Each definition/abbreviation can be expanded to a full description simply pointing the mouse cursor.

An example of the web downloadable "rest and stress parameters" electronic sheet is shown in Figure 9.

## Results

### The rest and stress data calculator algorithms

**Figure 10:**
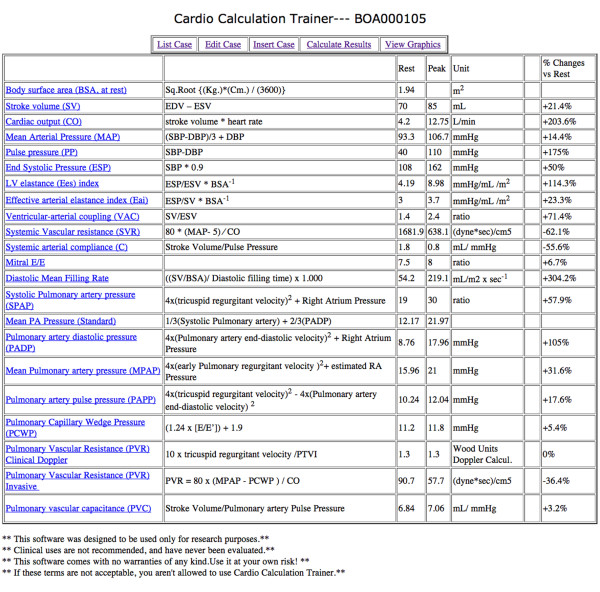
**Implementation of the computational training software**. Once linked to http://cctrainer.ifc.cnr.it., after filling the "insert case sheet" and clicking on the "rest and stress data calculator" button, the calculation results sheet opens. Calculation algorithms are shown in column 2 and software calculated results appear in the third "rest" and in the fourth "peak "column". In the sixth column the trainer will find % rest-peak change values. Each definition/abbreviation can be expanded to a full description simply pointing the mouse cursor.

A complete data set with software automatically calculated parameters, method of calculation and measure units is shown in Table 5. An example of the "calculated results" electronic sheet is shown in Figure 10. The specific algorithms are listed below:

**Table 5:** Rest and stress algorithm set

Calculated values	Method	*Measure unit*
**Body Surface Area (BSA, at rest)**	Sq.Root {(Kg.)*(Cm.)/(3600)}	*M^2^*
(rest, and peak stress)		
**Stroke Volume (SV)**	EDV - ESV	*mL*
**Cardiac Output (CO)**	stroke volume * heart rate	*L/min*
**Mean Arterial Pressure (MAP)**	(SBP-DBP)/3 + DBP	*mmHg*
**Pulse Pressure (PP)**	SBP-DBP	*mmHg*
**End Systolic Pressure (ESP)**	SBP * 0.9	*mmHg*
**LV Elastance (*E*_es_) index**	ESP/ESV * BSA ^-1^	*mmHg/mL/m^2^*
**Effective arterial elastance index (*E*ai)**	ESP/SV * BSA ^-1^	*mmHg/mL/m^2^*
**Ventricular-Arterial Coupling (VAC)**	*E*_es_/*E*a = (ESP/ESV)/(ESP/SV) = SV/ESV	*ratio*
**Systemic Vascular Resistance (SVR)**	80 * (MAP- 5) ⁄ Cardiac output	(dyne*sec)/cm^5^
**systemic arterial Compliance (C)**	Stroke Volume/Pulse Pressure	*mL/mmHg*
**Mitral E/e'**		*ratio*
**Diastolic Mean Filling Rate**	(SVi/Cardiological Diastolic Time) × 1,000	*mL/m^2 ^× sec^-1^*
**Diastolic time/systolic time ratio**	M-Mode or Sensor	*ratio*
**And, for right heart function:**		
**Pulmonary Artery Systolic Pressure (PASP)**	4×(Tricuspid regurgitant velocity)^2 ^+ Right Atrium Pressure	*mmHg*
**Pulmonary Artery Diastolic Pressure (PADP)**	4×(Pulmonary artery end-diastolic velocity)^2 ^+ Right Atrium Pressure	*mmHg*
**Mean PA Pressure(MPAP)**	1/3(SPAP) + 2/3(PADP).	*mmHg*
**Mean PA Pressure (MPAP)**	4×Peak pulmonary regurgitant velocity^2 ^	*mmHg*
**Pulmonary Artery Pulse Pressure (PAPP)**	4×(Tricuspid regurgitant velocity)^2 ^- 4×(Pulmonary artery end-diastolic velocity)^2^	*mmHg*
**Pulmonary Vascular Resistance (PVR)**	PVR = 80 × (MPAP - PCWP)/CO	(dyne*sec)/cm^5^
**Pulmonary Vascular Resistance (PVR)**	10 × Tricuspid regurgitant velocity/PTVI	*Woods units* X 80 = (dyne*sec)/cm^5^
**Pulmonary Vascular Capacitance (PVC)**	Stroke Volume/Pulmonary Artery Pulse Pressure	*mL/mmHg*

SBP = Systolic blood pressure; DBP = Diastolic blood pressure; TR = Tricuspid regurgitation; EDV = End-diastolic volume; ESV = End-systolic volume; RV = Right ventricular; PCWP = Pulmonary capillary wedge pressure; PTVI = Pulmonary time velocity integral; MPAP = Mean pulmonary artery pressure; SPAP = Systolic pulmonary artery pressure; PADP = Pulmonary artery diastolic pressure; MAP = Mean arterial pressure; SV = Stroke volume

Body Surface Area (BSA, m^2^)

BSA = (kg^0.5378^)(cm^0.3964^)

BSA = Sq. Root {(Kg.)*(Cm.)/(3600)}

Normal values 1.5-3.5 m^2 ^(adults)

BSA often used to standardize various physiologic measurements or dosing regimens

Stroke Volume (SV, mL)

The stroke volume is calculated as:

Stroke volume (SV, mL) = End-diastolic volume - End-systolic volume

(normal values 60-100 ml/beat)

Stroke Volume is indexed by dividing it by body surface area [31].

Stroke volume index (SVI, mL/m^2^) = Stroke volume/BSA

Cardiac Output (CO, L/min)

The Cardiac Output is calculated as:

Cardiac output = Heart rate * Stroke volume

Normal Cardiac Output is approx. 4-6 L/min

The relative contribution of end-systolic vs end-diastolic LV volume changes to increase stroke volume during stress can be calculated [32,33].

Cardiac Index (CI, L/min/m^2^)

Cardiac Index standardizes Cardiac Output for body size:CI = CO/BSA Normal Cardiac Index is approx. 2.5-3.6 L/min/m^2^

Mean Arterial Pressure (MAP, mmHg)

Mean Arterial Pressure (MAP, mmHg) = (SBP-DBP)/3 + DBP

Normal Mean Arterial Pressure is 70-100 mm Hg

Pulse Pressure (PP, mmHg)

Pulse pressure (mmhg) = systolic blood pressure (SBP) - diastolic blood pressure (DBP)

**Left Ventricular End-Systolic Pressure **(**LVESP, mmHg)**

LVESP = 0.9 * Systolic Blood Pressure (mmHg)

The most accurate estimate of LV End-Systolic Pressure is 0.9 * Systolic Blood Pressure [34]. Several aortic pressures have been proposed as surrogates for LV End-Systolic Pressure in an attempt to simplify the clinical assessment of arterial elastance (*E*_a_) and ventricular elastance (*E*_es_). Mean pressure, [35-37] mean ejection pressure, and the (2Systolic Blood Pressure + Diastolic Blood Pressure)/3 formula [38] significantly underestimated LV End-Systolic Pressure, the bias being higher in hypertensive patients than in normotensive subjects in all cases. Conversely, 0.9 * Systolic Blood Pressure gave a reliable estimate of LV End-Systolic Pressure whatever the prevailing aortic pressure. Previous results showed that *1*) peak LV pressure is usually achieved close to the volume point of minimal LV volume [39]*2*) peak LV pressure and LV End-Systolic Pressure are close in magnitude, although they occur at different points in time [40] and *3*) LV peak systolic pressure can be used instead of LV End-Systolic Pressure to calculate LV elastance with reasonably good accuracy [41]. The main clinical implication is that 0.9Systolic Blood Pressure may provide the most accurate estimate of LV End-Systolic Pressure, and this may improve the noninvasive calculation of ventricular elastance and arterial elastance (*E*_es _and *E*_a_). Other empirical LV End-Systolic Pressure estimates must not be used, especially in hypertensive patients. Importantly, the 0.9Systolic Blood Pressure approximation applies strictly to central pressure recordings and not to brachial artery pressure, given the physiological increases in systolic pressure observed from the aorta to periphery (pulse wave amplification phenomenon) [42].

#### End-Systolic Pressure-Volume determination (LVESP/ESV, mmHg/mL) or Left Ventricular Systolic Elastance, (*E*_es, _mmHg/mL)

The LVESP/ESV (or end-systolic elastance, *E*_es_) is determined as the ratio of the end systolic pressure/end-systolic volume. Because end-systolic volume varies directly with body size, end-systolic elastance can be indexed for body surface area (*E_esI _*= *E_es_*/BSA, mmHg/mL/m^2^) to better reflect differences with age and between the genders adjusted for differences in body size [43-45].

#### Arterial Elastance (*E*a, LVESP/SV, mmHg/mL)

Arterial elastance is essential to characterize the distal impedance of the arterial system downstream of the aortic valve. Arterial elastance is calculated as the ratio of end-systolic pressure (0.9SBP) by stroke volume, and expressed in the same units as Systolic Pressure/End Systolic Volume = mmHg/mL. Because stroke volume (and input impedance) varies directly with body size, arterial elastance can be adjusted for body surface area (*E*a_I_) to better reflect differences in arterial properties with age and between the genders adjusted for differences in body size [46]. This index models the arterial system as if it were an elastance receiving ejected volume from the heart and is affected principally by systemic vascular resistance, heart rate, and arterial compliance. Arterial elastance is elevated in heart failure, mainly because of tachycardia, increased systemic vascular resistance, and a reduction in systemic arterial compliance, compromising optimal coupling between the left ventricle and the arterial system.

#### Ventricular-Arterial Coupling (VAC, ratio)

Ventricular arterial coupling is indexed by the ratio of left ventricular systolic elastance index (*E*_es_, systolic pressure/end-systolic volume) to arterial elastance (*E*a, ratio of end-systolic pressure by stroke volume). Of note, ventricular-arterial coupling is ventricular elastance/arterial elastance, which can further be described as: end-systolic pressure/end-systolic LV volume divided by end-systolic pressure/stroke volume: the pressure terms in the numerator and the denominator cancel out, and ventricular-arterial coupling equals stroke volume/end-systolic volume. The noninvasive values of ventricular-arterial coupling are not dependent on blood pressure measurements and can be regarded as relatively accurate [47].

At rest, in healthy individuals, ventricular arterial coupling is maintained within a narrow range, which allows the cardiovascular system to optimize energetic efficiency at the expense of mechanical efficacy. During exercise, an acute mismatch between the arterial and ventricular systems occurs, due to a disproportionate increase in ventricular systolic elastance index *E*_esI _(from an average of 4.5 to 14.5 mmHg/mL/m^2^) vs arterial elastance *E*_aI _(from an average of 2.3 to 3.2 mmHg/mL/m^2^), to ensure that sufficient cardiac performance is achieved to meet the increased energetic requirements of the body. As a result ventricular arterial coupling (*E*_esI_/*E*_aI_) increases from an average of 1.9 to 4.5 in normals [48].

Changes in left ventricular-arterial coupling in DCM patients can be accounted for by both the absence of increase in the inotropic state of the left ventricle and by an increase in arterial elastance index. In DCM patients, ventricular systolic elastance index is almost equal to half of the arterial elastance index. Tachycardia exacerbates a baseline suboptimal ventricular-arterial coupling. As a result, blunted rest ventricular arterial coupling (*E*_esI_/*E*_aI_) does not significantly increase from an average of 0.4 to 0.5 in DCM patients [48].

##### Systemic Vascular Resistance (SVR, dyne*sec*cm^-5^)

SVR is calculated according to the traditional formula:

SVR=80*(MAP−5)/CO

where 5 is an approximation of the right atrial pressure and MAP is mean arterial pressure.

Vascular resistance is a term used to define the resistance to flow that must be overcome to push blood through the circulatory system. The resistance offered by the peripheral circulation is known as the systemic vascular resistance. The systemic vascular resistance may also be referred to as the total peripheral resistance. Vasoconstriction (i.e., decrease in blood vessel diameter) increases systemic vascular resistance, whereas vasodilatation (increase in diameter) decreases systemic vascular resistance [49]. Units for measuring vascular resistance are dyne*s*cm^-5 ^or Pascal seconds per cubic meter (MPa·s/m³) [50]. Pediatric cardiologists use hybrid reference units (HRU), also known as Wood units, as they were introduced by Dr. Paul Wood.

To convert from dyne*s*cm^-5 ^to Pascal seconds per cubic meter (MPa·s/m^3^), divide by 8.

To convert from dyne*s*cm^-5 ^to Wood units, divide by 80.

Normal systemic vascular resistance is 900-1300 dyne*sec*cm^-5 ^(or 90-130 MPa·s/m^3^) (or 11-16 Wood units)

##### Systemic Arterial Compliance (C, mL/mmHg)

Systemic arterial compliance = Stroke Volume/systemic arterial Pulse Pressure;

where pulse pressure = systolic blood pressure - diastolic blood pressure. Compliance is related to distensibility and arterial diameter [51-53].

##### Mitral E/e' (ratio)

Using the septal E/è' ratio, a ratio < 8 is usually associated with normal LV filling pressures, whereas a ratio > 15 is associated with increased filling pressures. The E/e' ratio is among the most reproducible echocardiographic parameters to estimate the pulmonary capillary wedge pressure and is the preferred prognostic parameter in many cardiac conditions. Elevated filling pressures are the main physiologic consequence of diastolic dysfunction. Filling pressures are considered elevated when the mean pulmonary capillary wedge pressure is > 12 mmHg or when the LV end-diastolic pressure is > 16 mmHg. Using the septal E/e' ratio, a ratio < 8 is usually associated with normal LV filling pressures, whereas a ratio > 15 is associated with increased filling pressures [54,55].

##### Diastolic filling rate (mean, mL/m^2 ^*sec^-1^)

Diastolic mean filling rate [24] is calculated at rest and at peak stress as mitral filling volume (considered equivalent to the Stroke Volume index) divided by cardiological diastolic time (automatically sensor estimated) × 1,000

$$\text{Diastolic Mean Filling Rate}=(\text{Stroke Volume index}/\text{Cardiological DiastolicTime})\times \text{1},000\left(\text{mL}/{\text{m}}^{\text{2}}*{\text{sec}}^{-\text{1}}\right)$$

The mean filling rate increases threefold in normals and far less in patients from rest to peak exercise, suggesting a larger filling capacity in normals. The diagnosis of heart failure with normal ejection fraction requires evidence of diastolic left ventricular dysfunction, obtained non-invasively by tissue Doppler (E/e' > 15). However, interpretable E/e' are not always obtained, since the most common source of uninterpretable tracings is fusion of E and A velocities due to tachycardia. The combination of a cutaneous operator-independent force sensor and 3 D stress echo allows a highly feasible, rapid and informative assessment of mitral inflow rate, which could be impaired in presence of diastolic dysfunction and provide insight into a novel form - feasible at last! - of diastolic stress echocardiography.

##### Right Ventricular Systolic Pressure (RVSP, mmHg) and Systolic Pulmonary Artery Pressure (SPAP, mmHg)

Right ventricular systolic pressure can be reliably determined from peak tricuspid regurgitation jet velocity, using the simplified Bernoulli equation and combining this value with an estimate of the right atrium pressure:

$$\text{Right ventricular systolic pressure}=\text{ 4}\times {\left(\text{tricuspid regurgitant velocity}\right)}^{\text{2}}+\text{right atrium pressure}\;\left(\text{mmHg}\right)$$

or

$$\text{RVSP}=\text{4}{\left(\text{V}\right)}^{\text{2}}+\text{RA pressure},$$

where V is the peak velocity (in meters per second) of the tricuspid valve regurgitant jet, and RA pressure is estimated from inferior vena cava diameter and respiratory changes. In the absence of a gradient across the pulmonary valve or right ventricular outflow tract, systolic pulmonary artery pressure is equal to right ventricular systolic pressure. In cases in which right ventricular systolic pressure is elevated, obstruction at the level of the right ventricular outflow tract or pulmonary valve should be excluded, especially in patients with congenital heart disease or post-pulmonary valve surgery. Tricuspid regurgitation velocity is usually obtained with continuous wave Doppler from the RV inflow or the apical four-chamber view position. Tricuspid regurgitation velocity reflects the pressure difference during systole between the RV and the right atrium. Normal resting values are usually defined as a peak tricuspid regurgitation velocity of < 2.8 to 2.9 m/s or a peak systolic pressure of 35 or 36 mm Hg, assuming an right atrium pressure of 3 to 5 mm Hg [56]. This value may increase with age and increasing body surface area. Further evaluation of patients with dyspnea with estimated right ventricular systolic pressure > 40 mm Hg.

##### Pulmonary Artery Diastolic Pressure (PADP, mmHg)

Pulmonary artery diastolic pressure can be estimated from the velocity of the end-diastolic pulmonary regurgitant jet using the modified Bernoulli equation:

$$[\text{Pulmonary Artery Diastolic Pressure}=\text{4}\times {\left(\text{end}-\text{diastolic pulmonary regurgitant velocity}\right)}^{\text{2}}+\text{Right Atrium pressure}]\;\left(\text{mmHg}\right)$$

The end-diastolic velocity of pulmonary regurgitation reflects the end-diastolic pressure gradient between the pulmonary artery and the RV. Normally, the pulmonary regurgitation end-diastolic pressure gradient is less than 5 mmHg. An increase in this pressure gradient (> 5 mmHg) has been found to correlate with systolic dysfunction, diastolic dysfunction, increased brain natriuretic peptide, and decreased functional status [57].

##### Mean Pulmonary Artery Pressure (Standard formula, mmHg)

Once systolic and diastolic pressures are known, mean pressure may be estimated by the standard formula [58]:

Mean Pulmonary Artery Pressure=1/3(Systolic Pulmonary Artery Pressure)+2/3(Pulmonary Artery Diastolic Pressure).

##### Mean Pulmonary Artery Pressure ("Doppler" methodology, mmHg)

The Mean Pulmonary Artery Pressure can also be estimated as:

$$\text{4}\times {\left(\text{early Pulmonary Regurgitant velocity}\right)}^{\text{2}}+\text{estimated Right Atrium pressure}\;\left(\text{mmHg}\right)$$

Whenever possible, it is helpful to use several methods to calculate mean pressure so that the internal consistency of the data can be challenged and confirmed.

##### Pulmonary Artery Pulse Pressure (PAPP, mmHg)

$$\text{Pulmonary Artery Pulse Pressure}=\text{4}\times {\left(\text{Tricuspid Regurgitant velocity}\right)}^{\text{2}}-\text{4}\times {\left(\text{Pulmonary Artery end}-\text{diastolic velocity}\right)}^{\text{2}}\;\left(\text{mmHg}\right)$$

##### Pulmonary Vascular Resistance (PVR, measurement via invasive monitoring, dyne*sec*cm^-5^)

Traditionally, Pulmonary Vascular Resistance is obtained by cardiac catheterization, using the following formula:

$$\text{Pulmonary Vascular Resistance}=\text{8}0\times \left(\text{mean PA pressure}-\text{PCWP}\right)/\text{CO}\;(\text{dyne}*\text{sec}*{\text{cm}}^{-\text{5}})$$

where mean PA pressure = Mean Pulmonary Arterial Pressure and PCWP = Pulmonary CapillaryWedge Pressure

Normal Pulmonary Vascular Resistance is 100-200 dyn·s*cm^-5 ^(or 10-20 MPa·s/m^3^) (or 1.25-2.5 Wood units)

Units for measuring vascular resistance are dyne*·s·*cm^-5 ^or pascal seconds per cubic meter (Pa·s/m³). Pediatric cardiologists use hybrid reference units (HRU), also known as Wood units, as they were introduced by Dr. Paul Wood. To convert from Wood units to MPa·s/m^3^, multiply by 8; or to dyne*s*cm^-5^, multiply by 80 [59]. The pressures are measured in units of millimeters of mercury (mmHg) and the cardiac output is measured in units of liters per minute (L/min).

##### Pulmonary Vascular Resistance (PVR, measurement via noninvasive Doppler echocardiography, Wood units)

Doppler echocardiography may provide a reliable, noninvasive method for determining Pulmonary Vascular Resistance:

$$\text{Pulmonary Vascular Resistance}\left(\text{Wood units}\right)={\text{TRV/TVI}}_{\text{RVOT}}*\text{1}0$$

Were TRV = peak tricuspid regurgitant velocity (m/s)

And TVI _RVOT = _right ventricular outflow tract time-velocity integral (cm)

To convert from Wood units to MPa·s/m^3^, multiply by 8; to dyne*·s*cm^-5^, multiply by 80. This relationship is not reliable in patients with very high Pulmonary Vascular Resistance, with measured Pulmonary Vascular Resistance > 8 Wood units. An elevation in systolic pulmonary artery pressure does not always imply an increased Pulmonary Vascular Resistance, as can be seen from the relationship whereby Δpressure = flow × resistance. A normal invasively measured Pulmonary Vascular Resistance is < 1.5 Wood units (< 120 dyne*·s*cm^-5^), and for the purpose of clinical studies in pulmonary hypertension, significant pulmonary hypertension is defined as a Pulmonary Vascular Resistance > 3 Wood units (> 240 dyne*·s*cm^-5^). Pulmonary Vascular Resistance distinguishes elevated pulmonary pressure due to high flow from that due to pulmonary vascular disease. The estimation of Pulmonary Vascular Resistance may be considered in subjects in whom pulmonary systolic pressure may be exaggerated by high stroke volume or misleadingly low (despite increased Pulmonary Vascular Resistance) by reduced stroke volume. The noninvasive estimation of Pulmonary Vascular Resistance should not be used as a substitute for the invasive evaluation of Pulmonary Vascular Resistance when this value is important in order to guide therapy [60-62].

##### Pulmonary Vascular Capacitance (PVC, mL/mmHg)

Pulmonary Vascular Capacitance=Stroke Volume/Pulmonary Artery Pulse Pressure (mL/mmHg)

Pulmonary vascular capacitance is a measure of the workload on the right ventricle, and pulmonary vascular capacitance determined by cardiac catheterization is a strong predictor of survival in patients with pulmonary hypertension. Pulmonary Vascular Capacitance can be derived noninvasively from a comprehensive 3 D and Doppler echocardiogram. Using the peak systolic tricuspid regurgitation velocity and the end-diastolic pulmonary regurgitation velocity, the modified Bernoulli equation is used to calculate the Pulmonary Artery systolic and diastolic pressures, and the Pulmonary Artery pulse pressure (Pulmonary Artery Pulse Pressure = Pulmonary Artery Systolic Pressure - Diastolic Pulmonary Artery Pressure, mmHg). Stroke volume is obtained using the volumetric flow through the left ventricular outflow tract, or by end-diastolic volume minus end-systolic volume. The novel measure of Pulmonary Vascular Capacitance, as determined by Doppler echocardiography, is a strong noninvasive predictor of mortality in patients with Pulmonary Hypertension and adds prognostic value to conventional risk markers. Mahapatra studied the prognostic value of pulmonary vascular capacitance determined by Doppler echocardiography in patients with pulmonary arterial hypertension and showed that in quartile analysis the lowest Pulmonary Vascular Capacitance quartile had a 4-year mortality of 39% whereas the highest Pulmonary Vascular Capacitance quartile had a mortality of 7% [63-65].

##### Measurement of Systolic Pulmonary Artery Pressure during exercise (SPAP, mmHg)

In normal subjects, exercise increases the stroke volume while the pulmonary vascular resistance decreases. Normal values are defined by Systolic Pulmonary Artery Pressure < 43 mmHg during exercise [27,66]. In well-trained athletes or those aged > 55 years, systolic Pulmonary Artery pressure as high as 55 to 60 mmHg at peak exercise is encountered. A pulmonary hypertensive response during exercise can be clinically important in several conditions, including valvular heart disease, heart failure, and Pulmonary Hypertension. From the pathophysiologic viewpoint, on the basis of the fundamental equation of flow (Flow = ΔPressure/Resistance), the abnormal exercise-induced increase in pressure can be ascribed to supranormal cardiac output (eg, in athletes) or to a normal increase in flow but a rise in resistance due to limited capability of the pulmonary vascular bed (eg, in chronic obstructive pulmonary disease or congenital heart disease) [27,67]. In this setting, the ratio of Δpressure (estimated by Tricuspid Regurgitation velocity) to flow (estimated by the RVOT time-velocity integral, or by the formula heart rate * stroke volume) may be helpful in distinguishing whether the increased pressure is related to an increase in flow or in resistance [27]. In patients with dyspnea of unknown etiology, with normal resting echocardiographic results and no evidence of coronary artery disease, it is reasonable to perform stress echocardiography to assess stress-induced Pulmonary Hypertension [27]. This technique should be considered as well in subjects with conditions associated with Pulmonary Hypertension. Supine bicycle exercise is the preferred method for Systolic Pulmonary Artery Pressure measurement. An upper limit of 43 mm Hg should be used in patients at non-extreme workloads. In subjects with valvular heart disease, the American College of Cardiology and American Heart Association cutoffs should be used to help guide management [27].

##### Pulmonary Capillary Wedge Pressure (PCWP, mmHg)

Pulmonary artery wedged pressure (also called pulmonary artery occlusion pressure or PAOP) is a measurement in which one of the pulmonary arteries is occluded, and the pressure downstream from the occlusion is measured in order to approximately sample the left atrial pressure. The catheter is threaded from peripheral vein into vena cava right atrium, right ventricle, pulmonary artery, and finally "wedged" with a balloon in a branch of the pulmonary artery. Because the catheter blocks flow, the pressure looks at the venous end of pulmonary circulation. Pulmonary Capillary Wedge Pressure estimates left atrial pressure.

Normal values:

Right atrial pressure. Mean value near 0 mmHg.

Right ventricle. Systolic = 25 mmHg. Diastolic = 0 mmHg.

Pulmonary artery. Mean value about 15 mmHg. Systolic about 25 mmHg. Diastolic about 8 mmHg.

Left atrial pressure. Mean about 5 mmHg.

An estimation of pulmonary capillary wedge pressure can be noninvasively assessed using the ratio of transmitral E velocity to the early diastolic mitral annulus velocity (e'), with the formula: Pulmonary Capillary Wedge Pressure (PCWP) = (1.24 × [E/e']) + 1.9 (mmHg)

Elevated filling pressures are the main physiologic consequence of diastolic dysfunction. Filling pressures are considered elevated when the mean pulmonary capillary wedge pressure is >12 mmHg or when the LV End-Diastolic Pressure is >16 mmHg. Using the septal E/e´ ratio, a ratio < 8 is usually associated with normal LV filling pressures, whereas a ratio >15 is associated with increased filling pressures [54,55].

#### Right Ventricular Diastolic Dysfunction

A growing number of acute and chronic conditions have been associated with RV diastolic dysfunction, including both pressure and volume overload pathologies, primary lung disease, ischemic heart disease, congenital heart disease, cardiomyopathies, LV dysfunction (via ventricular interdependence), systemic diseases, and the physiologic aging process [27]. The parameters used to assess RV diastolic function are essentially the same as those used to assess the left side. The ones that have been most validated are Doppler velocities of the transtricuspid flow (E, A, and E/A), tissue Doppler velocities of the tricuspid annulus (E', A', E'/A'), deceleration time, and isovolumic relaxation time (IVRT, msec). The tricuspid E/E' ratio, Right Atrium area or volume, and diastolic strain rate appear promising and have attracted interest in recent studies. Because of its thin wall, the right ventricle is very sensitive to afterload (wall stress), and it is also sensitive to changes in preload. The tricuspid E/E' ratio and Right Atrium volume have been shown to correlate well with hemodynamic parameters. Measurement of RV diastolic function should be considered in patients with suspected RV impairment as a marker of early or subtle RV dysfunction, or in patients with known RV impairment as a marker of poor prognosis. Grading of RV diastolic dysfunction should be done as follows: tricuspid E/A ratio < 0.8 suggests impaired relaxation, a tricuspid E/A ratio of 0.8 to 2.1 with an E/E' ratio > 6 or diastolic flow predominance in the hepatic veins suggests pseudonormal filling, and a tricuspid E/A ratio > 2.1 with a deceleration time < 120 ms suggests restrictive filling (as does late diastolic antegrade flow in the pulmonary artery). Further studies are warranted to validate the sensitivity and specificity and the prognostic implications of this classification.

## Testing

To test the WEB downloadable software for training in noninvasive cardiovascular hemodynamics, anonymous standard stress echo reports with baseline and stress measurements were submitted to 10 skilled echocardiographers (American Society of Echocardiography class III). Each echocardiographer was asked to calculate hemodynamic parameters starting from the sets of rest and stress measurements with two modalities (Table 6). A first set of calculations (Mode 1) was made by simply using personal knowledge of hemodynamic formulas and/or helping themselves with standard text books of cardiology. A second set of calculations (Mode 2) was made by using the web downloadable software. In both modalities (Mode 1 and Mode 2) the times needed for calculation and exactness of calculations were computed and compared. The time of calculation was 55 ± 63 min in mode 1 and 2 ± 0.5 min in mode 2 (p < 0.05 vs Mode 1). The exactness of calculations was 75 ± 20% in mode 1 and 100 ± 0% in mode 2 (p < 0.01 vs Mode 1).

**Table 6:** Testing the web downloadable software

Cardiologist #	1	2	3	4	5	6	7	8	9	10
**Mode 1 (subjective knowledge)**										
Time for calculation (min)	20	21	9	15	9	120	115	190	22	29
Exactness of calculations (exact/wrong, % of exactness)	34/6	32/8	16/24	24/16	28/12	38/2	40/0	40/0	22/18	26/14
	85%	80%	40%	60%	70%	95%	100%	100%	55%	65%
**Mode 2 (WEB software)**										
Time for calculation (min)	2	1,5	1,6	2	3	2	2	2,5	1,3	1,7

Exactness of calculations (exact/wrong, % of exactness)	40/0	40/0	40/0	40/0	40/0	40/0	40/0	40/0	40/0	40/0
	100%	100%	100%	100%	100%	100%	100%	100%	100%	100%

## Implementation

**Figure 11:**
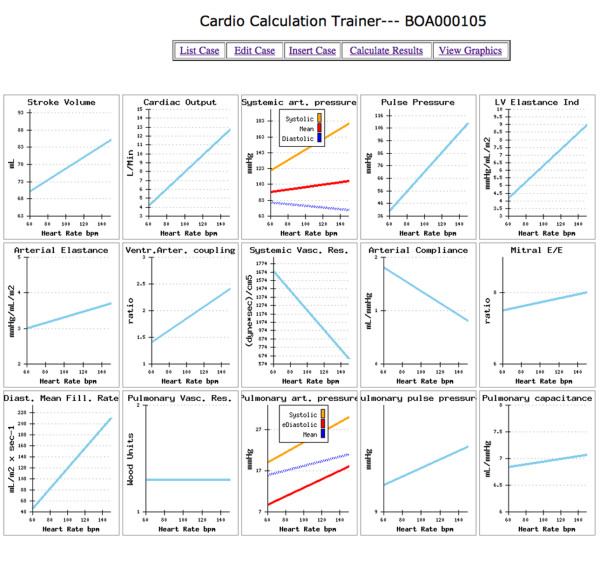
**Training example with a "normal subject" data set**. Clicking on the "view graphics" button opens the graph results sheet. For each calculated variable rest-peak changes are graphically displayed with heart rate values on the x-axis and variable value on the y-axis. LV elastance index at peak stress is nearly twice as large as arterial elastance index, with ventricular-arterial coupling normally set toward higher left ventricular work efficiency. Stroke volume increases through the use of the Frank-Starling mechanism and heart rate. Systemic vascular resistances drop markedly at peak exercise.

**Figure 12:**
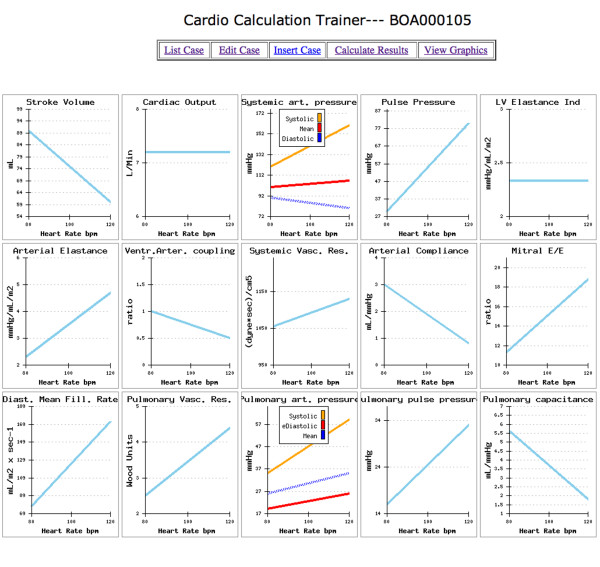
**Training example with a "DCM subject" data set**. Clicking on the "view graphics" button opens the graph results sheet. For each calculated variable rest-peak changes are graphically displayed with heart rate values on the x-axis and variable value on the y-axis. LV elastance index is markedly lower at peak exercise. At peak stress ventricular elastance index is less than one-half of the arterial elastance index, which results in decreased ventricular-arterial coupling and work efficiency. DCM patients display a vasoconstrictive response in peripheral circulation, with a modest and inconsistent decrease of SVR and a more marked drop of arterial compliance at peak stress.

The informatics infrastructure is available on the web, linking to http://cctrainer.ifc.cnr.it. Algorithms and data sheets can be freely downloaded by the WEB. Measures and indexes are obtained automatically by software processing. The exporting data in excel or SPSS format is possible for shorten calculations and to store data. Examples of the web downloadable "rest and stress parameters" electronic sheet is shown in Figure 9 and an example of the "calculated results" electronic sheet is shown in Figure 10. Images of graphically displayed results in a normal and DCM subject are shown in Figure 11 and Figure 12.

## Discussion

Clinical use of modern 3 D echocardiography is boosted by the marked reduction in acquisition time and the unique possibility of online rendering on the ultrasound system. The ability to visualize a virtual 3 D surface in real time - although limited to a sector size of about 30 degrees - offers new insights into cardiac pathomorphology even in patients with arrhythmias. Analysis of wide-angle 3 D datasets (90 by 90 degree pyramidal shape) is possible by combining the 3 D information of several [2-6] consecutive heart cycles. Three-dimensional (3D) volumetric imaging has potential advantages in stress echocardiography, including the ability to provide an unlimited number of planes for analysis.

Combination with 3 D color Doppler data allows additional assessment of valvular function as well as determination of flow in the left ventricular outflow tract and across septal defects. Dedicated software systems and technologies have been developed based on high-performance computers designed for graphic handling of three-dimensional images by providing possibilities beyond those obtainable with 2 D echocardiography. Beyond this, echocardiography enables the cardiologist to measure and calculate over 30 different noninvasive parameters including cardiac chamber size, indices of left ventricular performance, and estimates of mean left atrial pressure. However, the entire procedure requires meticulous measurements and time-consuming calculations, particularly when some of the data must be corrected for heart rate or body size. Because of this, many busy noninvasive cardiac laboratories routinely calculate only a few selected parameters in most patients. This problem of calculation is strongly amplified in the stress echo lab, since the 30 different noninvasive parameters become 60 if we also have to calculate peak stress data, 90 if we want to calculate rest-peak stress changes, and even more if we want to graphically display results. Quantitative and accurate calculation of a set of parameters allowing a complete characterization of cardiovascular hemodynamic needs data to be implemented in formulas - and until now, little or nothing has been done to help cardiologists utilize these data practically in the everyday busy echocardiography lab. In this paper we have shown how a simple web downloadable calculation trainer could greatly simplify the use and speed of calculation of a complete set of cardiologic parameters.

## Limitations of the study

### 3 D stress echocardiography

The success of imaging is high with pharmacologic stress but the feasibility is not established with exercise stress. The lower spatial and temporal resolution of 3 D imaging and artifacts introduced by suboptimal subvolume integration are limitations of the current 3 D technique. The ability to provide more planes for analysis has not been clearly shown to improve the accuracy of stress echocardiography. However, 3 D imaging eliminates apical foreshortening, which is common with 2 D imaging, and may improve detection of apical wall motion abnormalities. Development of automated image registration, quantitative analysis techniques, and single beat acquisition is needed to fully exploit the potential of 3 D imaging in the stress laboratory [68,69].

### Doppler indexes of diastolic function

A potential limitation of this study is the lack of information regarding Doppler indexes of diastolic function or dysfunction during stress. Doppler echocardiography is the preferred method for non-invasive diastolic function assessment. In current practice, most diastolic function indexes are derived by visual inspection of transmitral E- and A-waves [70]. However during exercise E- and A-waves become difficult to separate and discern when the A-wave merges with the E-wave and covers more than two-thirds of the E-wave deceleration, which typically occurs at heart rate > 100 beats/min. Furthermore, details of E and A waves are not reliably discernable above 120 bpm due to noise and resolution limitations [71]. Despite diastolic heart failure currently accounts for more than 50% of all heart failure patients and stress echocardiography is useful for the evaluation of patients with dyspnoea of possible cardiac origin, difficulties exist in assessing variables that influence cardiac diastolic filling during exercise.

The E/e´ ratio has been applied for that objective. In subjects with normal myocardial relaxation, E and e´ velocities increase proportionally, and the E/e´ ratio remains unchanged or is reduced. However, in patients with impaired myocardial relaxation, the increase in e´ with exercise is much less than that of mitral E velocity, such that the E/e´ ratio increases. In that regard, E/e´ was shown to relate significantly to LV filling pressures during exercise, when Doppler echocardiography was acquired simultaneously with cardiac catheterization [72,73].

In cardiac patients, mitral E velocity increases with exertion and stays increased for a few minutes after the termination of exercise, whereas e´ velocity remains reduced at baseline, exercise, and recovery. Therefore, E and e´ velocities can be recorded after exercise, after 2 D images have been obtained for wall motion analysis. However, the paucity of clinical data and the potential limitations in patients with regional LV dysfunction, mitral valve disease, and atrial fibrillation preclude recommendations for its routine clinical use at this time [54,55].

The combination of a cutaneous operator independent force sensor and 3 D stress echo allows a highly feasible, fast and informative assessment of mitral inflow rate, which could be impaired in presence of diastolic dysfunction and provide insight on a novel form of diastolic stress echocardiography [24].

### Diastolic Mean Filling Rate calculation

For the calculation of the Diastolic Mean Filling Rate we utilized the automatically sensor estimated cardiological diastolic time that overestimates the diastolic filling time. In fact, the cardiological diastolic time comprises diastolic filling time, the A_2 _- Mitral Opening interval (isovolumic relaxation) and the beginning of isovolumic contraction (mitral valve closure occurs with a definite albeit short delay after the start of the LV contraction) (Table 2). This certainly introduces an approximation; nevertheless, any error is systematically distributed from rest to peak stress, probably not affecting the slope values of this novel form of diastolic stress echocardiography. Filling volume is the numerator and the cardiological diastolic time is the denominator of the equation. An overestimation of diastolic filling time (in the presence of increased isovolumic relaxation time or of increased pre-ejection time) strengthens the clinical information given to a larger filling capacity at peak stress.

## Conclusions

After training one should be able to 1) list several functional parameters of the heart and vascular system at rest and during stress; 2) give typical values for systolic, diastolic and mean pressures in the systemic and pulmonary arterial system; 3) calculate arterial elastance from pressure and volume values, and know the steady (systemic vascular resistance) and pulsatile (systemic arterial compliance) components of the arterial elastance; 4) calculate left ventricular systolic elastance and the interaction of the LV with the arterial system, termed ventricular-arterial coupling, that is a central determinant of cardiovascular performance and cardiac energetics; 5) calculate pulmonary vascular resistance and pulmonary vascular capacitance; 6) calculate patterns of diastolic function and dysfunction at rest and during stress.

## Abbreviations

A_2_: aortic valve closure, aortic component of second sound; ASE: American Society of Echocardiography; BSA: Body surface area; C: Systemic arterial compliance; CO: Cardiac output; CI: Cardiac index; DBP: Diastolic blood pressure; *E*a: Effective arterial elastance; *E*es: Left ventricular elastance; EF: Ejection fraction; ESP: End systolic pressure; IVC: Inferior vena cava; IVCT: Isovolumic contraction time; IVRT: Isovolumic relaxation time; LV: Left ventricle; M_1 _: mitral component of first sound at time of mitral valve closure; MAP: Mean arterial pressure; P_2 _: pulmonary component of second sound; PA: Pulmonary artery; PADP: Pulmonary artery diastolic pressure; PAPP: Pulmonary artery pulse pressure; PCWP: Pulmonary capillary wedge pressure; PH: Pulmonary hypertension; PP: Pulse pressure; PVR: Pulmonary vascular resistance; PVC: Pulmonary vascular capacitance; RA: Right atrium; RV: Right ventricle; RVOT: Right ventricular outflow tract; RVSP: Right ventricular systolic pressure; SBP: Systolic blood pressure; SPAP: Systolic pulmonary artery pressure; SV: Stroke volume; SVR: Systemic vascular resistance; T_1 _: tricuspid valve closure, second component of first heart sound; 3 D: Three-dimensional; TR: Tricuspid regurgitation; TVI: Time velocity integral; 2 D: Two-dimensional; VAC: Ventricular arterial coupling.

## Competing interests

The authors declare that they have no competing interests.

## Authors' contributions

TB conceived this study, developed the SW system, performed the data analysis, and drafted the manuscript; DC participated in the development of the web system; GA contributed to data discussion; EPi contributed to the preparation of study design, data discussion, and critical revision of the manuscript. All authors read and approved the final manuscript.
